# Recovery following Thyroxine Treatment Withdrawal, but Not Propylthiouracil, Averts In Vivo and Ex Vivo Thyroxine-Provoked Cardiac Complications in Adult FVB/N Mice

**DOI:** 10.1155/2017/6071031

**Published:** 2017-07-16

**Authors:** Nancy S. Saad, Steven J. Repas, Kyle Floyd, Paul M. L. Janssen, Mohammad T. Elnakish

**Affiliations:** ^1^Department of Physiology and Cell Biology, College of Medicine, The Ohio State University, Columbus, OH, USA; ^2^Dorothy M. Davis Heart & Lung Research Institute, The Ohio State University, Columbus, OH, USA; ^3^Department of Pharmacology and Toxicology, Faculty of Pharmacy, Helwan University, Cairo, Egypt

## Abstract

Persistent cardiovascular pathology has been described in hyperthyroid patients even with effective antithyroid treatment. Here, we studied the effect of a well-known antithyroid drug, propylthiouracil (PTU; 20 mg/kg/day), on thyroxine (T4; 500 *µ*g/kg/day)-induced increase in blood pressure (BP), cardiac hypertrophy, and altered responses of the contractile myocardium both in vivo and ex vivo after 2 weeks of treatment. Furthermore, the potential recovery through 2 weeks of T4 treatment discontinuation was also investigated. PTU and T4 recovery partially reduced the T4-prompted increase in BP. Alternatively, PTU significantly improved the in vivo left ventricular (LV) function with no considerable effects on cardiac hypertrophy or ex vivo right ventricular (RV) contractile alterations subsequent to T4 treatment. Conversely, T4 recovery considerably enhanced the T4-provoked cardiac changes both in vivo and ex vivo. Altogether, our data is in agreement with the proposal that hyperthyroidism-induced cardiovascular pathology could persevere even with antithyroid treatments, such as PTU. However, this cannot be generalized and further investigation with different antithyroid treatments should be executed. Moreover, we reveal that recovery following experimental hyperthyroidism could potentially ameliorate cardiac function and decrease the risk for additional cardiac complications, yet, this appears to be model-dependent and should be cautiously construed.

## 1. Introduction

The heart is a well-known target of thyroid hormones. Hyperthyroidism may intensify antecedent heart disorder; nonetheless, it could also induce new cardiovascular diseases such as pulmonary hypertension [1], atrial fibrillation [2], and congestive heart failure [3]. Possible development of different cardiac pathologies ranging from physiologic cardiac hypertrophy with enhanced function [4] to cardiac dilation and heart failure [5] has been demonstrated in rodent hearts. Similarly, hyperthyroid patients could progress high-output heart failure with amplified heart contractility and cardiac output [6, 7], in addition to an augmented LV mass because of eccentric cardiac hypertrophy [8]. On the other hand, low-output heart failure has been also stated to occur in hyperthyroid patients [9], where cardiac output is dropped, systemic vascular resistance is enlarged, and LV contractility and LV filling are diminished, along with congestive heart failure signs and symptoms linked to dilated cardiomyopathy [3, 9–11]. Dilated cardiomyopathy coupled with LV systolic dysfunction is merely infrequently declared in hyperthyroid patients [3]. Besides, LV systolic dysfunction has been presented in only 50% of hyperthyroid patients with congestive heart failure comparable to congestive heart failure patients of nonthyroid origin; further mechanisms such as impaired LV diastolic function could be in charge of the congestive heart failure development in the remaining patients [3, 9, 12].

Cardiovascular complications have been revealed to significantly contribute to the death of hyperthyroid patients. Thus, early, efficient management of cardiac manifestations is essential for these patients. Yet, the heart failure treatment in hyperthyroid patients is challenging as it may be related to various entities [9]. Additionally, regardless the insight that hyperthyroidism is adjustable and deprived of long standing concerns, a substantial cardiovascular mortality in hyperthyroid patients even after effective treatment has been reported [9, 10, 13–15].

So far, inconsistent data has been conveyed on the cardiovascular outcomes following euthyroidism restoration in hyperthyroid patients either by effective antithyroid therapy [3, 10, 11] or by thyroidectomy [16, 17]. Similarly, changeable records have been presented in experimental animals with hyperthyroidism or with dilated cardiomyopathy associated with local hyperthyroidism subsequent to thyrostatic treatment [18, 19] or to recovery after cessation of exogenous T4/T3 (triiodothyronine) administration [20, 21]. However, we never studied theses effects in a mouse model of T4-induced cardiomyopathy that we have recently characterized. In this model, a T4 dose of 500 *µ*g/kg/day in the adult FVB/N male mice for 2 weeks leads to hypertension, cardiac hypertrophy, LV systolic dysfunction, and contractile changes in the isolated RV papillary muscles [22–24]. Therefore, the goal of the present study was to examine the effect of a well-known antithyroid drug, PTU, as well as the impact of recovery following the cessation of exogenous T4 administration on the T4-induced hemodynamic and cardiac complications in this model.

## 2. Material and Methods

### 2.1. Animals

Adult (9-10 months old) FVB/N male mice were purchased from the Jackson Laboratory (ME, USA) and kept at the Research Animal Facility of The Ohio State University for the whole duration of the experimental procedures. The experimental procedures and protocols used in this study were approved by the Animal Care and Use Committee of the Ohio State University, conforming to the Guide for the Care and Use of Laboratory Animals published by the United States National Institutes of Health (National Institutes of Health publication No. 85–23, revised 1996). A total of 38 mice were used for all experiments as described below.

### 2.2. Experimental Design

Basal BP as well as in vivo LV dimension and contractile function were assessed in 19 untreated mice. Sodium-L-thyroxine, T4, from Sigma-Aldrich (MO, USA) was prepared as described previously [25] and injected intraperitoneally (IP) in the 19 mice at a once-daily dose of 500 *µ*g/kg/day for 2 weeks [22–24, 26]. At the end of the 2 weeks T4 treatment, BP as well as in vivo LV dimension and contractile function were assessed again in the whole mouse number. Nine mice were then sacrificed; heart muscles were excised and processed for further ex vivo experiments in the T4-treated mice. However, the remaining 10 mice were left without T4 treatment for additional 2 weeks to test the potential recovery. Following these 2 weeks of T4 usage recovery, BP as well as in vivo LV dimension and contractile function were assessed and the mice were sacrificed for ex vivo experiments in the recovery group at that time. Basal heart weight (HW) plus basal contractile parameters of the ex vivo experiments were determined in additional 8 untreated mice. On the other hand, PTU from Cayman Chemical (MI, USA) was dissolved in dimethyl sulfoxide (DMSO), freshly diluted with PBS (final DMSO concentration was 5%), and administered by IP injection to 11 mice along with T4. PTU was administered at a pharmacological dose of 20 mg/kg/day. This dose has been revealed to result in powerful prohibiting upshots on T4 to T3 peripheral conversion in rodents, and corresponding dose has been exposed to turn out similar outcomes in human, as reported before [18]. At the end of the treatment period, BP as well as in vivo LV dimension and contractile function were assessed and the mice were sacrificed for ex vivo experiments at that point. Here, we did not use the DMSO (5%) in a separate group of mice based on our previous data [23, 24] showing that DMSO up to 10% has no significant impact on the T4-induced cardiovascular complications in this model.

### 2.3. Blood Pressure (BP) Measurements

BP was measured noninvasively in conscious untrained mice by the tail cuff method using a six-Channel CODA High Throughput Acquisition system (Kent Scientific Corporation, Torrington, CT, USA) as described before [24]. Each experimental session consisted of 10 acclimatization cycles followed by 10 BP measurement cycles. The accepted cycles as identified by the BP measurement software only are included. The average of accepted cycles from one session was used for systolic BP (SBP), diastolic BP (DBP), and mean arterial pressure (MAP) in each mouse.

### 2.4. Echocardiography

By using a high-frequency ultrasound imaging system (VEVO 2100, Visual Sonics, Toronto, ON, Canada) the in vivo LV dimension and contractile function in mice were evaluated as described before [22–25]. Experimental mice were anesthetized with isoflurane at a concentration of 3% and then maintained at 1.5% isoflurane using nasal prongs during the whole procedure. The measurements were taken from the parasternal short-axis view in M-mode to view the LV movement during systole and diastole corresponding to the electrocardiogram. All data and imaging were analyzed by the Visual Sonics Cardiac Measurements Package.

### 2.5. Heart Weight, Cardiac Muscle Preparation, and Experimental Setup

First, mice were weighed, and five minutes after IP heparin injection mice were euthanized by cervical dislocation. After bilateral thoracotomy, hearts were rapidly excised and placed in Krebs–Henseleit buffer containing (in mmol/L): 120 NaCl, 5 KCl, 2 MgSO_4_, 1.2 NaH2PO_4_, 20 NaHCO_3_, 0.25 Ca^2+^, and 10 glucose, equilibrated with 95% O_2_- 5% CO_2_, resulting in a pH of 7.4. Additionally, 20 mmol/L 2,3-butanedione monoxime (BDM) was added to the dissection buffer to prevent cutting injury [22–24]. Extra noncardiac tissues, such as fat and pieces of lung, were carefully removed. After hearts were blotted gently on Kimwipes they rapidly transferred to a small weigh dish that contained clean oxygenated Krebs–Henseleit/BDM buffer that was tarred to zero on an electronic analytical balance to get the exact wet HW. Heart/body weight (BW) ratios were then calculated and expressed as mg/g. Subsequently, hearts were carefully opened and repeatedly perfused with the same oxygenated Krebs–Henseleit/BDM buffer, and blood was thoroughly washed out. From the RV, uniform linear papillary muscles were carefully dissected. The dimensions of muscles were measured using a calibration reticule in the ocular of the dissection microscope (40x, resolution ~ 10 *µ*m). The cross-sectional areas were calculated assuming ellipsoid cross-sectional shapes. There was no significant difference between average dimensions (width × thickness × length) of the Basal (0.31 ± 0.04 × 0.21 ± 0.03 × 0.87 ± 0.14 mm), T4 (0.28 ± 0.02 × 0.19 ± 0.01 × 0.68 ± 0.11 mm), PTU + T4 (0.27 ± 0.02 × 0.18 ± 0.01 × 0.81 ± 0.11 mm), and recovery (0.28 ± 0.02 × 0.19 ± 0.01 × 0.87 ± 0.10 mm) groups. *P* values are 0.9760 (Kruskal-Wallis analysis), 0.9760 (Kruskal-Wallis analysis), and 0.6568 (ordinary one-way analysis of variance [ANOVA]), respectively.

With the use of the dissection microscope, muscles were mounted between basket-shaped extension of a force transducer (KG7, Scientific Instruments, Heidelberg, Germany) and a hook (valve end) connected to a micromanipulator as previously mentioned [22–24]. Muscles were superfused with the same buffer at 37.5°C as above (with the exception that BDM was omitted) and stimulated at 4 Hz. Extracellular Ca^2+^ concentration was raised to 2 mmol/L and muscles were allowed to stabilize for at least 30 minutes before the experimental protocol was initiated. The 4 Hz baseline was selected rather than a more physiological 12 Hz, as in our previous reports [22–24]. However, to study more physiological frequencies, 12 Hz contractions were also assessed, but only for brief periods. Generally, muscles were stretched to an optimal length where a small increase in length resulted in nearly equal increases in resting tension and active developed tension. This length was selected to be comparable to the maximally attained length in vivo at the end of diastole [27].

To obtain a broad scope of quantitative data to dissect contractile function and dysfunction, two of the three main mechanisms utilized in vivo to physiologically modify force of contraction, frequency-dependent activation, and *β*-adrenergic stimulation were assessed in mouse papillary muscles under near physiological conditions as previously mentioned [22–24]. We assessed the effect of increasing stimulation frequencies between 4 and 14 Hz, spanning the entire in vivo range of the mouse. At each frequency, forces were allowed to reach steady state before data were recorded. The effects of *β*-adrenergic stimulation were assessed by a concentration–response curve with isoproterenol (10^−9^–10^−6^ mol/l) at a baseline stimulation frequency of 4 Hz.

In all experiments performed peak isometric developed force (*F*_dev_) was determined and normalized to the cross-sectional area of the muscle. Additionally, as a force-independent parameter of force decay kinetics, time to peak force (TTP) and time from peak force to 50% relaxation (RT50) were determined. Muscles with an initial *F*_dev_ or a *F*_dev_ after restabilization following frequency-dependent activation < 5 mN/mm^2^ were excluded from the analysis of all experimental parameters. Additionally, muscles that displayed arrhythmia early at the initial isoproterenol concentrations were excluded from the final isoproterenol-*F*_dev_ data analysis.

### 2.6. Data Analysis and Statistics

Generally, a two-tailed value of *P* ≤ 0.05 was considered statistically significant. Data are presented as mean ± SEM and were analyzed by either ordinary one-way ANOVA or repeated measures ANOVA followed by Tukey-Kramer's post hoc multiple comparison test. Ordinary one-way ANOVA assumes that the data are sampled from populations that follow Gaussian distributions. This assumption was tested using Kolmogorov and Smirnov test, which showed that all data sampled passed normality test. Also, it assumes that the data are sampled from populations with identical SDs. This assumption was tested using Bartlett test, which suggested that the differences among the SDs of the groups were not significant, except in few cases. In these exceptional cases, Kruskal-Wallis test (nonparametric ANOVA) was applied followed by Dunn's post hoc multiple comparison test. On the other hand, ordinary repeated measures ANOVA assumes effective matching among means. If the matching appears not to be effective, Friedman test (nonparametric repeated measures ANOVA) was applied followed by Dunn's post hoc multiple comparison test.

## 3. Results

In agreement with our previous data [22–24], T4 treatment significantly increased the mouse DBP, SBP, and MAP in comparison to the basal group (*P* < 0.001) (Table 1). PTU treatment partially decreased the T4-induced increases in the DBP (*P* = 0.2119), SBP (*P* = 0.0678), and MAP (*P* = 0.1446) to levels that also are not significantly different from those of the basal group (*P* = 0.0952, 0.1380, and 0.1038, resp.) (Table 1). Similarly, the 2-week T4 recovery moderately reduced the T4-provoked increases in the DBP (*P* = 0.1634), SBP (*P* = 0.0647), and MAP (*P* = 0.1152) to levels that are insignificant compared to those of the basal group too (*P* = 0.1659, 0.1894, and 0.1716, resp.) (Table 1).

**Figure 1:**
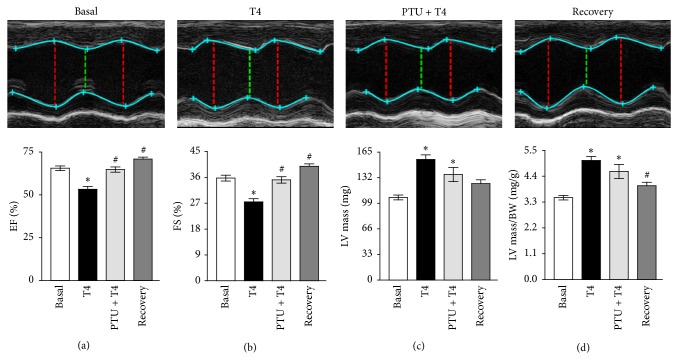
Echocardiography analysis of mouse hearts. (a) Ejection fraction (EF), (b) fractional shortening (FS), (c) left ventricle (LV) mass, and (d) left ventricle (LV) mass/body weight (BW) ratio. Basal; *n* = 19, thyroxin (T4); *n* = 19, propylthiouracil (PTU) + T4; *n* = 11, recovery; *n* = 10. ^**∗**^A significant change compared to basal, ^#^a significant change compared to T4. EF and FS; one-way ANOVA followed by Tukey-Kramer's post hoc multiple comparison test. LV mass and LV mass/BW ratio; Kruskal-Wallis test followed by Dunn's post hoc multiple comparison test.

Echocardiography analysis showed that T4 treatment resulted in a significant LV systolic dysfunction as manifested by considerably decreased ejection fraction (EF) (53.29 ± 1.70%; *P* < 0.001) (Figure 1(a)) and fractional shortening (FS) (27.47 ± 1.12%; *P* < 0.001) (Figure 1(b)) compared to those of the basal group (EF: 65.58 ± 1.34% and FS: 35.74 ± 1.02%), as we showed before [22–24]. Interestingly, PTU as well as the 2-week T4 recovery repressed the T4-induced LV systolic dysfunctions as indicated by significantly improved EF (PTU + T4: 64.79 ± 1.52% and recovery: 70.93 ± 1.09%; *P* < 0.001) (Figure 1(a)) and FS (PTU + T4: 35.12 ± 1.12 and recovery: 39.850 ± .8383%; *P* < 0.001) (Figure 1(b)). Besides, echocardiographic evaluation of cardiac dimensions exposed that T4 treatment led to substantial increases in LV mass (155 ± 6 mg; *P* < 0.001) (Figure 1(c)) and LV mass/BW ratio (5.07 ± 0.17 mg/g; *P* < 0.001) (Figure 1(d)) compared to the basal group (LV mass: 106 ± 3 mg, and LV mass/BW: 3.49 ± 0.10 mg/g), as we previously described [22–24]. Surprisingly, PTU treatment could not significantly decrease such T4-stimulated increases in LV mass (136 ± 9 mg) (Figure 1(c)) or LV mass/BW ratio (4.60 ± 0.30 mg/g) (Figure 1(d)) compared to the T4-treated mice and they still are significantly higher than those of the basal group (*P* < 0.05 and *P* < 0.01, resp.). Conversely, the 2-week T4 recovery clearly attenuated the T4-provoked increase in LV mass (124 ± 5 mg) (Figure 1(c)), but it did reach significance only in case of LV mass/BW ratio (3.99 ± 0.15 mg/g; *P* < 0.05) (Figure 1(d)) compared to the T4-treated mice. Further, this was not only insignificant compared to the basal group but also compared to the (PTU + T4)-treated mice (Figures 1(c) and 1(d)).

Assessment of morphological parameters showed that there is no significant difference in the BW of all groups at the end of the treatment period. Corresponding to our previous findings [22–24], T4 treatment expressively amplified both HW (*P* < 0.001) and HW/BW ratio (*P* < 0.001) compared to the basal group, which confirm the cardiac hypertrophy development in these mice (Table 2). Nevertheless, PTU treatment could not decrease either the T4-induced increase in HW or the HW/BW ratio compared to the T4-treated mice and they still are significantly higher than those of the basal group (*P* < 0.001 and *P* < 0.001, resp.) (Table 2). Oppositely, the 2-week T4 recovery significantly decreased the T4-induced increases in HW (*P* < 0.001 and *P* < 0.01) and HW/ BW ratio (*P* < 0.001 and *P* < 0.001) compared to both T4-treated mice and (PTU + T4)-treated mice, respectively, (Table 2).

**Figure 2:**
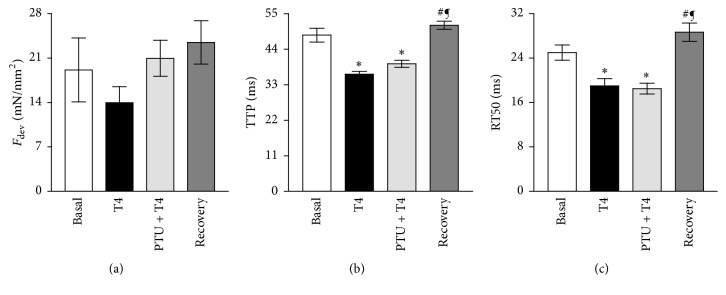
Contractile profile of isolated right ventricular papillary muscles. (a) Mean active isometric developed force (*F*_dev_), (b) their corresponding time to peak (TTP), and (c) 50% relaxation time (RT_50_) at 4 Hz stimulation frequency and 2 mmol/L Ca^2+^. Basal; *n* = 8, thyroxin (T4); *n* = 8, propylthiouracil (PTU) + T4; *n* = 10, recovery; *n* = 9. ^**∗**^A significant change compared to basal, ^#^a significant change compared to T4, and ^¶^a significant change compared to PTU + T4. One-way ANOVA followed by Tukey-Kramer's post hoc multiple comparison test.

In the direction of illustrating the prospective deficits in cardiac contractile strength, we examined the contractile performance of the isolated RV papillary muscles. The length-dependent activation (Frank-Starling mechanism) is well-maintained in the hearts of these T4-treated mice as we have lately defined [23]. Thus, we only studied the effects of frequency-dependent activation (Bowditch effect) and adrenergic stimulation (fight/flight response) on these isolated muscles. Under proximate physiological temperature and at a preload leading to sarcomere length around the in vivo end-diastolic values of 2.2 *μ*m [27], *F*_dev_ of RV papillary muscles was sustained (*P* = 0.3039) among the groups at a stimulation frequency of 4 Hz (Basal: 19 ± 5 mN/mm^2^; T4: 14 ± 3 mN/mm^2^; PTU + T4: 21 ± 3 mN/mm^2^ and recovery: 24 ± 3 mN/mm^2^) (Figure 2(a)). Contrariwise, muscles from T4-treated mice exhibited faster contraction and relaxation as shown by significantly declined TTP (36 ± 1 ms; *P* < 0.001) (Figure 2(b)) and RT50 (19 ± 1 ms; *P* < 0.05) (Figure 2(c)) compared to those of the basal group (TTP: 48 ± 2 ms and RT50: 25 ± 1 ms). Treatment with PTU was not able to significantly change the T4-induced drop in the TTP (40 ± 1 ms) (Figure 2(b)) or the RT50 (19 ± 1 ms) (Figure 2(c)), and they still are significantly lower than those of the basal group (TTP: *P* < 0.001 and RT50: *P* < 0.01). However, 2-week T4 recovery returned the T4-induced decrease in TTP (51 ± 1 ms; *P* < 0.001 versus T4 and PTU + T4 groups) (Figure 2(b)) and RT50 (29 ± 2 ms; *P* < 0.001 versus T4 and PTU + T4 groups) (Figure 2(c)) back to the basal levels, respectively.

**Figure 3:**
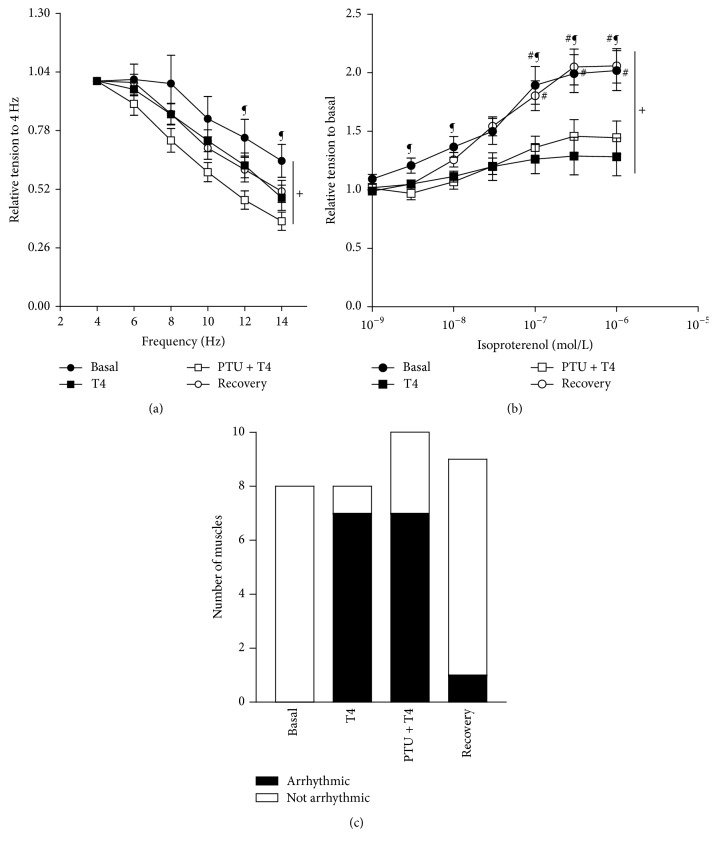
(a) Force-frequency relationship of isolated right ventricular papillary muscles. Isometric developed force values are expressed as a fraction of its corresponding value at the basal frequency of 4 Hz and presented as mean ± SEM. Basal; *n* = 8, thyroxin (T4); *n* = 8, propylthiouracil (PTU) + T4; *n* = 10, recovery; *n* = 9. ^¶^A significant change compared to PTU + T4. One-way ANOVA followed by Tukey-Kramer's post hoc multiple comparison test at all frequencies except the 8 Hz (Kruskal-Wallis analysis). ^+^A significant change compared to both the group and frequency variation (Friedman nonparametric repeated measures ANOVA followed by Dunn's post hoc multiple comparison test). (b) *β*-Adrenergic stimulation of isolated right ventricular papillary muscles. Isometric developed force values following *β*-adrenergic stimulation are expressed as a fraction of its corresponding value at the basal frequency of 4 Hz before isoproterenol addition and presented as mean ± SEM. (c) Arrhythmic activity at 1 mmol/L of isoproterenol. Basal; *n* = 8, thyroxin (T4); *n* = 7, propylthiouracil (PTU) + T4; *n* = 9, recovery; *n* = 9. ^#^A significant change compared to T4, and ^¶^a significant change compared to PTU + T4. Significance mark above the error bar represents the group of the upper symbol, while a significance mark on the side reflects the lower. One-way ANOVA followed by Tukey-Kramer's post hoc multiple comparison test at all isoproterenol concentrations, except the 10^−9^ and 3 × 10^−9^ M (Kruskal-Wallis test followed by Dunn's post hoc multiple comparison test). ^+^A significant change compared to both the group and isoproterenol concentration variations (repeated measures ANOVA followed by Tukey-Kramer's post hoc multiple comparison test).

Aimed at a fewer indefinite extrapolation to the in vivo consequence, *F*_dev_ was estimated within the murine in vivo physiological range (8–12 Hz) in addition to the baseline stimulation frequency of 4 Hz. Unexpectedly, one-way ANOVA showed insignificant differences among basal, T4, or recovery groups in response to increasing frequencies at all frequency range. Nevertheless, PTU treatment along with T4 resulted in a significantly negative response to increasing frequency at 12 Hz and 14 Hz (*P* < 0.01) compared to the basal group (Figure 3(a)). Alternatively, repeated measures ANOVA showed a significant effect for both the group (*P* < 0.001) and frequency (*P* < 0.001) variations on the *F*_dev_. However, there was no significant interaction between the group and frequency variations. Additionally, the *β*-adrenergic stimulation effect was evaluated by a concentration–response curve with isoproterenol (10^−9^–10^−6^ mol/l) at a baseline stimulation frequency of 4 Hz. As we previously demonstrated [22–24], one-way ANOVA revealed that under full *β*-adrenergic stimulation (1 *μ*mol/l isoproterenol), muscles from T4-treated mice displayed markedly depressed responses (*P* < 0.05) versus those from the basal group (Figure 3(b)). PTU treatment could not recover this blunted isoproterenol response in the T4-treated mice and it still clearly blunted compared to the basal group (Figure 3(b)). However, the 2-week T4 recovery returned the T4-evoked blunted isoproterenol response back to the basal level, which is significantly higher than those of the T4 (*P* < 0.01) and PTU + T4 (*P* < 0.05) groups (Figure 3(b)). In addition, repeated measures ANOVA showed a significant effect for both the group (*P* < 0.001) and isoproterenol concentration (*P* < 0.001) variations on the *F*_dev_. Likewise, there was a significant interaction between the 2 variants (*P* < 0.001), as we previously verified [22–24]. Moreover, at full *β*-adrenergic stimulation (1 *μ*mol/l isoproterenol): 7 out of 8 muscles from T4 group and 7 out of 10 muscles from PTU + T4 group showed arrhythmic behavior versus 1 out of 9 muscles from the recovery group and none out of 8 muscles in the basal group (Figure 3(c)).

## 4. Discussion

Overt and subclinical hyperthyroid patients are at increased cardiac mortality risk. This excessive risk could be due to increased atrial arrhythmias as well as heart failure in these patients [9]. Actually, euthyroidism leads to a prompt clinical enhancement of cardiac function and congestive heart failure symptoms. Yet, in some cases heart failure may come to be irreversible [3]. In this regard, it has been reported that a quick medical enhancement represented by improved LV function and attained sinus rhythm in a hyperthyroid patient following restoration of euthyroidism by thyrostatic treatment [11]. Conversely, some cardiovascular complications have been confirmed to persist in overt hyperthyroid patients in spite of active antithyroid treatment [3, 10]. Also, it has been indicated that full thyroidectomy may reinstate euthyroidism, thus refining heart function in patients of amiodarone-induced thyrotoxicosis with serious LV dysfunction [16]. Though, other studies revealed that hyperthyroidism advances the hospitalization risk attributable to cardiovascular diseases and the risk continued up to two decades even after effective thyroidectomy [17]. Similar to the discrepancy in the human clinical data, Wang et al. proved that antithyroid treatment (PTU) in ΔK210 mutant mice with dilated cardiomyopathy accompanied with local hyperthyroidism attenuated the cardiac hypertrophy and enhanced the LV function and life expectancy of these mice [19]. However, active thyrostatic treatment (PTU) has been oppositely shown to be unable to prevent the T4-induced hypertrophy in hyperthyroid rats [18]. Additionally, cessation of T3 treatment for 3 weeks (recovery) following 3 weeks of T3 treatment has been shown to inhibit the T3-induced LV dysfunction in adolescent (~2 months old), but not in adult (~6 months old) C57Bl/6N female mice. This presented a tendency of continuous cardiac dysfunction in these adult mice even after T3 recovery [20]. In the same way, Hoefig et al. attested that a recovery from a 2-week thyrotoxicosis in male C57BL/6J mice (3-4 months old) resulted in a significant decrease in the T4-stimulated increase in HW, yet, HW/BW did not reach significance. Still, mRNA levels of* Hcn2*, *β*2-adrenergic receptor, and* Bnf*, a thyroid hormone-dependent ventricular hypertrophy marker, were raised, along with severe bradycardia, concluding that thyrotoxicosis may have pathological consequences that could continue ahead of the serum T4 level recovery [21]. However, we never investigated theses effects in a mouse model of T4-induced cardiomyopathy that we have recently characterized [22–24]. To the best of our knowledge, the current study represents the first report that studies the effect of a well-known antithyroid drug, PTU, and T4 usage recovery not only on T4-induced hemodynamic alteration (e.g., BP) but also on cardiac hypertrophy and altered responses of the contractile myocardium both in vivo at the whole heart level and ex vivo at the cardiac tissue level.

PTU is a thiouracil derivative that is considered as one of the most commonly used antithyroid drugs in the hyperthyroidism management [28]. This is attributed to its ability to inhibit the thyroperoxidase, a key enzyme of thyroid hormone biosynthesis, and thus decrease the thyroid hormone production [29]. In addition, PTU is an iodothyronine deiodinase inhibitor, which inhibits the conversion of T4 to T3 in the peripheral tissues [18, 30]. Indeed, T3 is rated as the biologically effective hormone. The thyroid hormone receptors affinity for T3 is around tenfold greater than T4, which means that T4 should be transformed to T3 in order to generate strong thyroid hormone receptors-motivated outcomes [31]. This raised the option that the entire hormonal upshot of exogenously administered T4 in experimental models could be due to its peripheral transformation to T3 [32, 33]. Therefore, a remarkable diminution in hormonal effects of T4 would be expected if PTU repressed the T4 monodeiodination to T3 [33]. There are 2 types of the iodothyronine deiodinases, which convert the T4 to the bioactive form (T3) in the extrathyroidal tissues, including the iodothyronine deiodinase I (D1) and the iodothyronine deiodinase II (D2) [30]. A number of prior clinical studies have suggested that the D2 pathway is the main production source of extrathyroidal T3 in humans [30, 34, 35], and the expression of its messenger RNA has been shown to be higher in human than rodent heart [36, 37]. Yet, a recent study has demonstrated that local hyperthyroidism by transcriptional D2 gene upregulation could be a vital fundamental mechanism for the cardiac remodeling in dilated cardiomyopathy mouse model [19]. Interestingly, in this later study [19], the PTU, which is a selective D1 inhibitor [30], considerably prohibited the cardiac remodeling and expanded the mice survival. The authors suggested that decreasing the serum level of thyroid hormones by antithyroid drug may be efficient in inhibiting the sudden death and progression of heart failure in dilated cardiomyopathy [19]. Moreover, clinical studies proposed that D1 pathway becomes the predominant extrathyroidal source of T3 during hyperthyroidism as indicated by the rapid fall in serum T3 following the PTU treatment, a selective D1 inhibitor in hyperthyroid patients [38]. Thus, PTU has been used in the current study in order to examine its efficiency in preventing the T4-induced cardiovascular pathology in our model as reported before [18].

In accordance with our previous data [22–24] along with others data [39–41], T4 increased the mouse BP in the current study. PTU and T4 recovery moderately decreased such increase in the BP to levels that are not significantly different from those of the T4-treated or the basal group mice. Partial inhibitory effects of PTU and T4 recovery on increased BP following T4 treatment, particularly the SBP (*P* = 0.0678 and 0.0647, resp.), could be due to interaction with several pathways that have been reported to be involved in the development of hypertension, such as increased nitric oxide synthase [23, 39–41] and/or possible increase in corticosterone subsequent to hyperthyroidism [42, 43]. PTU has been reported to decrease the corticotrophin-stimulated levels of plasma corticosterone in rats [44]. Also, it has been shown to decrease neuronal nitric oxide synthase [45] that is reported as a major key player in the T4-induced hypertension [39]. Thus far, the exact mechanism needs to be explored.

Noteworthy, our data exhibited that PTU significantly prevented the T4-induced in vivo LV dysfunction and the T4 usage recovery markedly inhibited all T4-prompted cardiac pathologies, including cardiac hypertrophy, in vivo LV dysfunction, and ex vivo RV contractile changes; however, they could not distinctly avert T4-stimulated hypertension in these mice. We have previously revealed that T4-induced cardiac changes in this model are independent of hemodynamic changes, that is, increased BP, which means that T4-induced cardiac pathology and hemodynamic alterations could be produced through different signaling pathways [23]. A recent study reported that around 90% of definitely regulated hepatic target genes restored to basal expression levels after 10 days of T4 recovery from chronic hyperthyroidism (2-week T4 treatment) in mice; nonetheless, around 10% did not, even with serum thyroid hormone level normalization [46]. It has been concluded that serum thyroid hormone and thyroid stimulating hormone levels, which reveal the hypothalamic/pituitary/thyroid axis reliability and negative feedback, could not precisely represent the T3 receptiveness of definite tissues and their genes throughout chronic hyperthyroidism and the conversion from thyrotoxicosis to euthyroidism [46]. Furthermore, these results could assist elucidate the perseverance of physiological and metabolic changes, which are clinically detected in some patients in spite of serum thyroid function tests renormalization after treatment [47].

T4-induced cardiac hypertrophy is well recognized and occasionally associated with LV dysfunction [5, 20, 22–24] in conjunction with striking RV contractile complications as we presented before [22–24]. In line with these data, T4 administration in this study augmented the HW, HW/BW ratio, LV mass, and LV mass/BW ratio confirming the development of cardiac hypertrophy. In addition, it significantly declined the LV EF and FS triggering a substantial in vivo LV contractile dysfunction compared to the basal group mice. Equally, excised papillary muscles from the RV of T4-treated mice revealed the hypertrophied and dysfunctional heart signs, such as reduced contraction/relaxation times and blunted *β*-adrenergic response together with increased arrhythmia. The difference among the basal and T4 groups on the frequency-response was similar, not statistically significant. This response (reduced basal and T4 force at different frequency rates of stimulation) was smaller than in our previous studies, where the difference reached a statistical significance [22–24]. The exact reason for this inconsistency is unknown. The only difference between this study and the previous reports is the slight change in the mouse age; 9-10 months versus 7–9 months, respectively. This might have a slight effect on the observed force-frequency relationship. Formerly, we have revealed that isolated papillary muscles from the older wild-type FVB/N mice RV (12–14 months) displayed a slightly negative FFR [48]. Only, PTU + T4 treatment did result in a significantly more negative response to increasing frequency at 12 and 14 Hz compared to the basal group.

Here, we showed that PTU could not significantly attenuate the T4-induced cardiac hypertrophy, but it significantly improved the in vivo LV function as indicated by increased EF and FS compared to T4-treated mice. In agreement with our results, Wang et al. reported that PTU improved the cardiac function and life expectancy by increasing LV EF in dilated cardiomyopathy mice linked to local hyperthyroidism [19]. In contrast to our results, this later report showed that PTU also prevented the cardiac hypertrophic remodeling in these mice [19]. Yet, in a consistency with our data, Chopra et al. demonstrated that PTU with the exact dose (20 mg/Kg/day) was not able to inhibit the T4-induced hypertrophy in hyperthyroid rats even though it returned the serum levels of the bioactive T3 back to normal [18]. Additionally, we established that PTU could not inhibit the T4-stimulated ex vivo RV contractile alterations, including TTP, RT50, blunted *β*-adrenergic response, or increased development of arrhythmia. Indeed, this is consistent with our previous data showing that apocynin, a NADPH oxidase inhibitor, only prevented the T4-induced LV dysfunction with no positive effects on either the cardiac hypertrophy or the ex vivo RV contractile changes [23]. PTU has been shown to partially inhibit thyroid NADPH oxidase activity in vitro [29]. Overall, our results show signs of being in agreement with the proposal that cardiovascular changes are common in hyperthyroid patients at presentation; nonetheless, some could continue even with actual antithyroid treatment, such as PTU [10]. Still, this cannot be generalized and further investigation with different antithyroid drugs should be executed. For example, in the same study that agrees with our data and showed that PTU could not inhibit the T4-induced cardiac hypertrophy, another antithyroid drug, sodium ipodate [49], was able to do it even though the serum T3 levels and other thyroid hormone peripheral effects were comparable in the 2 groups. This suggested that multiple sites in the heart may be involved in the thyroid hormone effects instead of a single site [18].

On the other hand, we showed that T4 recovery due to cessation of T4 treatment for equal period of 2 weeks in the adult mice used in this study almost prevented all T4-induced cardiac pathologies, including cardiac hypertrophy, in vivo LV dysfunction, and ex vivo RV contractile changes. Importantly, T4 treatment in mice for 2 weeks followed by 2 weeks [21] or 10 days [46] of withdrawal returned the bioactive T3 serum concentrations to near basal levels, which confirms the opportunity of restoring the euthyroid state after recovery. Our data possibly reveals that recovery of hyperthyroidism could ameliorate cardiac function and decrease the risk for further cardiac complications. In contrast, it has been shown that adult mice are missing this recovery prospective, presenting a tendency of continuous cardiac dysfunction [20]. The variation in the mouse species (FVB/N versus C57Bl/6N), sex (male versus female), and age (9-10 months versus ~6 months old) between this study and the later one [20] clearly indicates that effects of recovery on cardiac changes following experimental hyperthyroidism seem to be model-dependent and should be carefully interpreted.

Finally, the heart primarily depends on serum T3 due to the absence of a major myocyte intracellular activity of deiodinase, which converts T4 to T3, and it looks that T3, but not T4, is conveyed into the myocyte. T3 binds to thyroid hormone nuclear receptors and exerts its cellular actions [50]. But, it has been reported that this nuclear thyroid hormone-signaling pathway cannot entirely elucidate the extracellular T3 stimulating effects of the cardiac growth [18, 19]. In agreement with this proposal, our data showed that PTU, which works through inhibiting T4 conversion into T3, could not decrease T4-induced cardiac hypertrophy or ex vivo RV contractile manifestations, but the T4 withdrawal recovery did. This could confirm that the mechanisms responsible for T4-promoted cardiovascular changes are possibly due to mutual thyroid hormone effects on certain molecular pathways, which require further exploration.

## 5. Conclusions

In summary, our data is in agreement with the proposal that hyperthyroidism-induced cardiovascular pathology could persevere even with antithyroid treatments, such as PTU. However, this cannot be generalized and further investigation with different antithyroid treatments are warranted. Moreover, our data shows that recovery of experimental hyperthyroidism could potentially ameliorate cardiac function and decrease the risk for further cardiac complications. Yet, this appears to be model-dependent and should be cautiously construed. Finally, we propose that the mechanisms responsible for T4-promoted cardiovascular changes are possibly due to mutual thyroid hormone effects on certain molecular pathways, which require further exploration.

## Figures and Tables

**Table 1:** Blood pressure of mice.

	Basal	T4	PTU + T4	Recovery
DBP (mmHg)	95 ± 2	116 ± 3^*∗*^	106 ± 4	105 ± 5
SBP (mmHg)	122 ± 2	145 ± 3^*∗*^	133 ± 4	132 ± 5
MAP (mmHg)	104 ± 2	125 ± 3^*∗*^	115 ± 4	114 ± 5

Diastolic blood pressure (DBP), systolic blood pressure (SBP), and mean arterial pressure (MAB). Basal; *n* = 19, thyroxin (T4); *n* = 19, propylthiouracil (PTU) + T4; *n* = 11, recovery; *n* = 10. ^*∗*^A significant change compared to basal (one-way ANOVA followed by Tukey-Kramer multiple comparison test).

**Table 2:** Morphological data.

	Basal	T4	PTU + T4	Recovery
BW (g)	29 ± 0.9	31 ± 0.5	30 ± 0.7	31 ± 0.9
HW (mg)	134 ± 3	186 ± 6^*∗*^	180 ± 6^*∗*^	152 ± 4^#¶^
HW/BW (mg/g)	4.72 ± 0.10	5.91 ± 0.12^*∗*^	6.09 ± 0.16^*∗*^	4.88 ± 0.09^#¶^

BW: body weight, HW: heart weight. Basal; *n* = 8, thyroxin (T4); *n* = 9, propylthiouracil (PTU) + T4; *n* = 11, recovery; *n* = 10. ^*∗*^A significant change compared to basal, ^#^a significant change compared to T4, and ^¶^a significant change compared to PTU + T4 (one-way ANOVA followed by Tukey-Kramer multiple comparison test).

## References

[1] Siu C.-W., Zhang X.-H., Yung C. (2007). Hemodynamic changes in hyperthyroidism-related pulmonary hypertension: a prospective echocardiographic study. *The Journal of Clinical Endocrinology Metabolism*.

[2] Siu C.-W., Pong V., Zhang X. (2009). Risk of ischemic stroke after new-onset atrial fibrillation in patients with hyperthyroidism. *Heart Rhythm*.

[3] Siu C.-W., Yeung C.-Y., Lau C.-P., Kung A. W. C., Tse H.-F. (2007). Incidence, clinical characteristics and outcome of congestive heart failure as the initial presentation in patients with primary hyperthyroidism. *Heart*.

[4] Kuzman J. A., Vogelsang K. A., Thomas T. A., Gerdes A. M. (2005). L-Thyroxine activates Akt signaling in the heart. *Journal of Molecular and Cellular Cardiology*.

[5] Kuzman J. A., Thomas T. A., Vogelsang K. A., Said S., Anderson B. E., Gerdes A. M. (2005). Effects of induced hyperthyroidism in normal and cardiomyopathic hamsters. *Journal of Applied Physiology*.

[6] Cruz F. E. S., Cheriex E. C., Smeets J. L. R. M. (1990). Reversibility of tachycardia-induced cardiomyopathy after cure of incessant supraventricular tachycardia. *Journal of the American College of Cardiology*.

[7] deGroot W. J., Leonard J. J. (1970). Hyperthyroidism as a high cardiac output state. *American Heart Journal*.

[8] Marcisz C., Jonderko G., Wróblewski T., Kurzawska G., Mazur F. (2006). Left ventricular mass in patients with hyperthyroidism. *Medical Science Monitor*.

[9] Biondi B. (2012). Mechanisms in endocrinology: heart failure and thyroid dysfunction. *European Journal of Endocrinology*.

[10] Osman F., Franklyn J. A., Holder R. L., Sheppard M. C., Gammage M. D. (2007). Cardiovascular Manifestations of Hyperthyroidism Before and After Antithyroid Therapy. A Matched Case-Control Study. *Journal of the American College of Cardiology*.

[11] Dhadke S. V., Dhadke V. N. (2006). Reversible cardiomyopathy. *Journal of Association of Physicians of India*.

[12] Yue W.-S., Chong B.-H., Zhang X.-H. (2011). Hyperthyroidism-induced left ventricular diastolic dysfunction: implication in hyperthyroidism-related heart failure. *Clinical Endocrinology*.

[13] Brandt F., Green A., Hegedüs L., Brix T. H. (2011). A critical review and meta-analysis of the association between overt hyperthyroidism and mortality. *European Journal of Endocrinology*.

[14] Franklyn J. A., Maisonneuve P., Sheppard M. C., Betteridge J., Boyle P. (1998). Mortality after the treatment of hyperthyroidism with radioactive iodine. *The New England Journal of Medicine*.

[15] Hall P., Lundell G., Holm L.-E. (1993). Mortality in patients treated for hyperthyroidism with iodine-131. *Acta Endocrinologica*.

[16] Tomisti L., Materazzi G., Bartalena L. (2012). Total thyroidectomy in patients with amiodarone-induced thyrotoxicosis and severe left ventricular systolic dysfunction. *Journal of Clinical Endocrinology and Metabolism*.

[17] Ryödi E., Salmi J., Jaatinen P. (2014). Cardiovascular morbidity and mortality in surgically treated hyperthyroidism - A nation-wide cohort study with a long-term follow-up. *Clinical Endocrinology*.

[18] Chopra I. J., Huang T.-S., Hurd R. E., Solomon D. H. (1984). A study of cardiac effects of thyroid hormones: Evidence for amelioration of the effects of thyroxine by sodium ipodate. *Endocrinology*.

[19] Wang Y.-Y., Morimoto S., Du C.-K. (2010). Up-regulation of type 2 iodothyronine deiodinase in dilated cardiomyopathy. *Cardiovascular Research*.

[20] Hübner N. S., Merkle A., Jung B., von Elverfeldt D., Harsan L.-A. (2015). Analysis of left ventricular function of the mouse heart during experimentally induced hyperthyroidism and recovery. *NMR in Biomedicine*.

[21] Hoefig C. S., Harder L., Oelkrug R. (2016). Thermoregulatory and cardiovascular consequences of a transient thyrotoxicosis and recovery in male mice. *Endocrinology*.

[22] Elnakish M. T., Moldovan L., Khan M., Hassanain H. H., Janssen P. M. L. (2013). Myocardial Rac1 exhibits partial involvement in thyroxin-induced cardiomyocyte hypertrophy and its inhibition is not sufficient to improve cardiac dysfunction or contractile abnormalities in mouse papillary muscles. *Journal of Cardiovascular Pharmacology*.

[23] Elnakish M. T., Schultz E. J., Gearinger R. L. (2015). Differential involvement of various sources of reactive oxygen species in thyroxin-induced hemodynamic changes and contractile dysfunction of the heart and diaphragm muscles. *Free Radical Biology and Medicine*.

[24] Saad N. S., Floyd K., Ahmed A. A. E., Mohler P. J., Janssen P. M. L., Elnakish M. T. (2016). The effect of sorafenib, tadalafil and macitentan treatments on thyroxin-induced hemodynamic changes and cardiac abnormalities. *PLoS ONE*.

[25] Elnakish M. T., Hassona M. D. H., Alhaj M. A. (2012). Rac-induced left ventricular dilation in thyroxin-treated zmracd transgenic mice: role of cardiomyocyte apoptosis and myocardial fibrosis. *PLoS ONE*.

[26] Kuzman J. A., O'Connell T. D., Gerdes A. M. (2007). Rapamycin prevents thyroid hormone-induced cardiac hypertrophy. *Endocrinology*.

[27] Rodriguez E. K., Hunter W. C., Royce M. J., Leppo M. K., Douglas A. S., Weisman H. F. (1992). A method to reconstruct myocardial sarcomere lengths and orientations at transmural sites in beating canine hearts. *The American Journal of Physiology—Heart and Circulatory Physiology*.

[28] Nakamura H., Noh J. Y., Itoh K. (2007). Comparison of methimazole and propylthiouracil in patients with hyperthyroidism caused by Graves' disease. *The Journal of Clinical Endocrinology &amp; Metabolism*.

[29] Freitas Ferreira A. C., de Carvalho Cardoso L., Rosenthal D., Pires de Carvalho D. (2003). Thyroid Ca^2+^/NADPH-dependent H_2_O_2_ generation is partially inhibited by propylthiouracil and methimazole. *European Journal of Biochemistry*.

[30] Bianco A. C., Kim B. W. (2006). Deiodinases: implications of the local control of thyroid hormone action. *The Journal of Clinical Investigation*.

[31] Jabbar A., Pingitore A., Pearce S. H. S., Zaman A., Iervasi G., Razvi S. (2016). Thyroid hormones and cardiovascular disease. *Nature Reviews Cardiology*.

[32] Schwartz H. L., Surks M. I., Oppenheimer J. H. (1971). Quantitation of extrathyroidal conversion of L-thyroxine to 3,5,3'-triiodo-L-thyronine in the rat. *Journal of Clinical Investigation*.

[33] Oppenheimer J. H., Schwartz H. L., Surks M. I. (1972). Propylthiouracil inhibits the conversion of L-thyroxine to L-triiodothyronine. An explanation of the antithyroxine effect of propylthiouracil and evidence supporting the concept that triiodothyronine is the active thyroid hormone.. *Journal of Clinical Investigation*.

[34] Geffner D. L., Azukizawa M., Hershman J. M. (1975). Propylthiouracil blocks extrathyroidal conversion of thyroxine to triiodothyronine and augments thyrotropin secretion in man. *Journal of Clinical Investigation*.

[35] Saberi M., Sterling F. H., Utiger R. D. (1975). Reduction in extrathyroidal triiodothyronine production by propylthiouracil in man. *Journal of Clinical Investigation*.

[36] Croteau W., Davey J. C., Galton V. A., St. Germain D. L. (1996). Cloning of the mammalian type II iodothyronine deiodinase. A selenoprotein differentially expressed and regulated in human and rat brain and other tissues. *Journal of Clinical Investigation*.

[37] Dentice M., Morisco C., Vitale M., Rossi G., Fenzi G., Salvatore D. (2003). The different cardiac expression of the type 2 iodothyronine deiodinase gene between human and rat is related to the differential response of the dio2 genes to Nkx-2.5 and GATA-4 transcription factors. *Molecular Endocrinology*.

[38] Abuid J., Larsen P. R. (1974). Triiodothyronine and thyroxine in hyperthyroidism. Comparison of the acute changes during therapy with antithyroid agents. *Journal of Clinical Investigation*.

[39] Wangensteen R., Rodríguez-Gómez I., Moreno J. M., Álvarez-Guerra M., Osuna A., Vargas F. (2006). Effects of chronic treatment with 7-nitroindazole in hyperthyroid rats. *The American Journal of Physiology—Regulatory Integrative and Comparative Physiology*.

[40] Rodríguez-Gómez I., Sainz J., Wangensteen R. (2003). Increased pressor sensitivity to chronic nitric oxide deficiency in hyperthyroid rats. *Hypertension*.

[41] Rodríguez-Gómez I., Wangensteen R., Moreno J. M., Chamorro V., Osuna A., Vargas F. (2005). Effects of chronic inhibition of inducible nitric oxide synthase in hyperthyroid rats. *American Journal of Physiology—Endocrinology and Metabolism*.

[42] Vazir H., Whitehouse B. J., Vinson G. P., McCredie E. (1981). Effects of prolonged ACTH treatment on adrenal steroidogenesis and blood pressure in rats. *Acta Endocrinologica*.

[43] Johnson E. O., Kamilaris T. C., Calogero A. E., Gold P. W., Chrousos G. P. (2005). Experimentally-induced hyperthyroidism is associated with activation of the rat hypothalamic-pituitary-adrenal axis. *European Journal of Endocrinology*.

[44] Lo M.-J., Wang S-W., Kau M.-M. (1998). Pharmacological effects of propylthiouracil on corticosterone secretion in male rats. *Journal of investigative medicine: the official publication of the American Federation for Clinical Research*.

[45] Serfozo Z., Kiss P. B., Kukor Z. (2008). Thyroid hormones affect the level and activity of nitric oxide synthase in rat cerebral cortex during postnatal development. *Neurochemical Research*.

[46] Ohba K., Leow M. K.-S., Singh B. K. (2016). Desensitization and incomplete recovery of hepatic target genes after chronic thyroid hormone treatment and withdrawal in male adult mice. *Endocrinology*.

[47] Fahrenfort J. J., Wilterdink A. M. L., Van Der Veen E. A. (2000). Long-term residual complaints and psychosocial sequelae after remission of hyperthyroidism. *Psychoneuroendocrinology*.

[48] Elnakish M. T., Hassanain H. H., Janssen P. M. L. (2012). Vascular remodeling-associated hypertension leads to left ventricular hypertrophy and contractile dysfunction in profilin-1 transgenic mice. *Journal of Cardiovascular Pharmacology*.

[49] Chopra I. J., van Herle A. J., Korenman S. G., Viosca S., Younai S. (1995). Use of sodium ipodate in management of hyperthyroidism in subacute thyroiditis. *Journal of Clinical Endocrinology and Metabolism*.

[50] Klein I., Danzi S. (2007). Thyroid disease and the heart. *Circulation*.

